# Can federal policy help overcome systemically reinforced racial inequities in social determinants of health? An observational study of Georgia and neighboring U.S. states

**DOI:** 10.1186/s12889-024-17726-4

**Published:** 2024-01-26

**Authors:** Eric Napierala, Bill Rencher, Lori Solomon, Chris Parker

**Affiliations:** Georgia Health Policy Center, Andrew Young School of Policy Studies, Georgia State University, 55 Park Place, 8th floor, Atlanta, GA 30303 USA

**Keywords:** Health disparities, Inequities, Policy, Social determinants of health, Structural barriers, Systemic racism

## Abstract

**Background:**

Despite increasing attention to racial inequities in social determinants of health and health outcomes, less attention has been focused on how structural barriers — embedded in programs and codified in laws — shape opportunities to achieve health.

**Methods:**

To better understand how U.S. federal policies targets structural barriers to opportunity and health at the population level, we conducted a legal review to identify landmark pieces of federal policy that held potential to impact key social determinants of health. Then, using publicly available data for Georgia and five neighboring U.S. states (Alabama, Florida, North Carolina, South Carolina, and Tennessee), we conducted an observational case study to examine recent trends for access to health care, housing, and education because they were each associated with comprehensive federal legislation meant to alleviate inequities resulting from long-standing structural barriers and were each identified by Healthy People 2030 as key social determinants of health.

**Results:**

From 2010 to 2021, population-level improvements were seen in health insurance rates, mortgage and rental burden, and educational attainment, with improvements seen for both Black and White populations in Georgia, regionally in the Southeast region, and nationally in the United States. However, seemingly meaningful gaps between the Black and White populations across social determinants of health have not been eliminated at any geographical level.

**Conclusions:**

This analysis adds to a growing body of evidence that historically racialized social structures hamper Black populations’ opportunities to build wealth, gain a quality education, own a home in a neighborhood of opportunity, and access health care, compared to their White peers. Given that the root causes of health disparities and inequities lie at the intersection of health, health care, economics, education, and other social systems, a multisectoral approach to policy is needed to address these systemic issues. While federal laws do provide momentum for proximal benefits for social change, in modern federalism they alone are insufficient to address needed local system change and nonlegal policy interventions, implemented at the local programmatic level, may serve as complementary mechanism to address the lingering effects of barriers to equal opportunity.

## Background

In the United States, Black populations — living in both urban and rural areas — have been disproportionately affected by limited access to health care and worse health outcomes, compared to geographically similar White populations [1–3]. Similarly, racial disparities in income, employment, housing, educational opportunity, food availability, transportation, and incarceration have long existed [3, 4].

There is increasing recognition of the link between socioeconomic factors and health disparities and inequities. These social determinants of health are defined by the Centers for Disease Control and Prevention and the World Health Organization as the conditions in which people are born, grow, work, live, and age, and the wider set of forces and systems shaping the conditions of daily life (e.g., economic policies and systems, development agendas, social norms, social policies, racism, climate change, and political systems) [5].

As part of its data-driven approach to improve health and well-being, Healthy People 2030 groups the social determinants of health into five domains (economic stability, education access and quality, health care access and quality, neighborhood and built environment, and social and community context) and has related national outcome objectives for each [6].

### Systemic, structural barriers

Socioeconomic factors have driven racial inequities in health outcomes since the origin of the United States as a country [7]. Yet, no one health-promoting behavior, socioeconomic factor, or environmental exposure can explain the consistent pattern of racial health disparities, as they currently persist or as they have existed for centuries. Despite the increasing attention to social determinants of health, less attention has been focused on how the “wider set of forces and systems shaping the conditions of daily life” [5] (e.g., economic policies and systems, social policies, racism, and political systems) shapes health. These structural barriers — embedded in programs and codified in laws — have prevented equal opportunity for wealth, education, home ownership, access to health care, and general well-being across races.

Thus, racial health disparities sit within the context of persistent, historically racialized social structures that determine differential access to risks, opportunities, and resources that drive health [8]. There is growing evidence that these embedded social structures themselves are actually a driver of racial disparities in health beyond what is explainable by socioeconomic factors [8].

Our goal was to better understand how the impact of U.S. federal policies that broadly promote opportunity for health and well-being is experienced at the population level by race. We first conducted a legal review to identify landmark pieces of historical, federal policy that held potential to impact key social determinants of health. Then, using publicly available data for Georgia and five neighboring U.S. states, we conducted an observational case study to examine recent trends related to the impact of U.S. federal policy on racial disparities in select social determinants of health outcomes (access to health care, education, and housing). By assessing impact over time, we aim to improve understanding of how federal policy might support the alleviation of systemically promulgated racial inequities at the state population level, even when the policy is race neutral at face value.

## Methods

### Legal review

We searched the academic literature and law review articles to identify the most comprehensive pieces of federal policy (legislation or Supreme Court case) pertaining to nationally recognized social determinants of health — access to health care, education, and housing. Specific search terms included “housing and racial disparities and law,” “education and racial disparities and law,” and “health care and racial disparities and law.”

### Population-level outcomes for social determinants of health

Access to health care, housing, and education were selected as areas of focus because they were each associated with comprehensive federal legislation meant to alleviate inequities resulting from long-standing structural barriers and were identified by Healthy People 2030 [6] as key social determinants of health. For each, we identified at least two population-level measures: at least one directly measuring a problem addressed by federal policy and at least one captured in Healthy People 2030’s 10-year national objectives. Table 1 summarizes selected measures.

**Table 1:** Summary of selected data measures by social determinant of health and associated population level represented

Social Determinant of Health	Healthy People 2030 Measure [6]	Other Measure
Access to health care		
	Proportion of people with health insurance at the state level	Primary care Health Professional Shortage Area at the state level and Georgia county level
Housing		
	Proportion of families that spend > 30% income on housing at the state level	Homeownership at the state level
Education		
	Percent of population with a high school degree or higher at the state level	School enrollment by race at the Georgia county level

Comparisons across indices of social determinants were made at the state population level between Georgia and its five neighboring states (Alabama, Florida, North Carolina, South Carolina, and Tennessee), where comparable state-level data exists, as well as for Georgia versus the national level for measures, where comparable data existed.

#### Access to health care

We used two proxies for access to care — uninsurance rates (directly tied to remedies offered by the Affordable Care Act) and primary care provider shortages (a designation that aligns geographically with other established markers of medical, economic, and geographic vulnerability) [9]. Uninsurance rates serve as indicators of the ability to afford health care, while primary care Health Professional Shortage Areas (HPSAs) reflect the ability to access preventive care and are positively associated with health outcomes and the opportunity to achieve good health [10].

##### Uninsured rate

Uninsurance rates were determined at the state level and national level using data from the U.S. Census Bureau [11]. A person is considered uninsured if not covered by employer-provided insurance, privately purchased insurance, Medicare, Medicaid, or other governmental insurance (e.g., TRICARE, other military care, or Veterans Administration-provided insurance) [11]. The Census Bureau does not consider respondents to have coverage if their only coverage is from Indian Health Services, as these policies are not always comprehensive [11].

Uninsured rates were calculated at the state and national level for 2010 (pre-Affordable Care Act), 2015 (soonest reporting time period following implementation of the Affordable Care Act), and 2021 (the most recent year with available data). Rates were compared by Black and White race across states and compared to national rates. Health insurance rate was calculated by dividing the total number of individuals with health insurance by the total civilian noninstitutionalized population, respective of race. Percent change from prior time period was calculated by state and nationally for each race.

State-level primary care HPSA metrics were accessed via the Kaiser Family Foundation [12]. County-level primary care HPSA data was accessed via the Bureau of Health Workforce, Health Resources and Services Administration (HRSA), U.S. Department of Health and Human Services website [13]. The most current update data available (2018 to 2022) was used when accessed April 2023.

We relied upon HRSA definitions for geographic HPSA (shortage of providers for an entire group of people within a defined geographic area) and population HPSAs (a shortage of providers for a specific group of people within a defined geographic area; e.g., low-income populations, homeless populations, and migrant farmworker populations) [13].

#### Education

We used two measures as proxies for education — educational attainment and school enrollment by race (directly tied to remedies offered by *Brown v. Board of Education*). Educational attainment is a contemporary measure but may reflect the complex and lingering impacts of centuries of unequal investment in Black and White student education [14, 15]. School enrollment by race served as a proxy for segregation, which while legally remedied, is recognized to persist and impact student achievement [16–18].

##### Education attainment

Trends in education attainment (defined as percent of population with a high school degree equivalency or higher) were determined using data from the U.S. Census Bureau [11]. Comparisons were made across states and nationally, by Black and White race for 2010, 2015, and 2021. We used a different proxy for education than Health People 2030’s proportion of high school students who graduate in four years [6] to enable comparisons across state and national levels.

##### Public K-12 school enrollment demographics

As a proxy for racial segregation in schools, Georgia Department of Education data [19] was used to determine demographic breakdown of school enrollment by race for public schools for each county in the state at the start of the 2022–2023 school year. Comparisons were made for racial composition of Georgia public schools versus racial composition of the state population, overall, which relied on U.S. Census population estimates [20]. However, comparisons were not possible across states or nationally.

#### Housing

The two housing proxies used included: homeownership at the state level (directly tied to remedies offered by the Fair Housing Act) and housing burden (proportion of families that spend > 30% of income on housing) at the state level [21]. Both housing-related indices reflect the complex relationship between housing stability and financial well-being. Homeowners generally experience better physical health, mental health, and better access to health care, while a higher housing burden is associated with worse physical and mental health outcomes [22–26].

##### Homeownership

U.S. Census Bureau data [11] was used to assess temporal changes (2010, 2015, and 2021) in home ownership in Georgia, among neighboring states, and at the national level by Black and White race.

##### Mortgage and rental burden

Using data from the U.S. Census Bureau [11], comparisons in mortgage and rental burden (defined as > 30% of income spent on rent or mortgage) were made across Georgia, neighboring states, and nationally by Black and White race. Selected years included 2010, 2015, and 2021 to parallel comparisons across other social determinants of health.

## Results

### Legal review findings

An examination of federal law and policy related to the three key social determinants of health — access to health care, education, housing — identified the following pivotal federal policies.

- Access to health care
  o Hospital Survey and Construction Act of 1946 [Pub. L. 79–725]
  o Title VI of the 1964 Civil Rights Act [Pub. L. 88–352]
  o Patient Protection and Affordable Care Act [Pub. L. 111–148]
- Education
  o GI Bill of 1944 [Pub. L. 78–346]
  o *Brown v. Board of Education* [347 U.S. 483 (1954)]
- Housing
  o National Housing Act of 1934 [Pub. L. 73–479]
  o Fair Housing Act, Title VIII of the 1968 Civil Rights Act [Pub. L. 90–284]

Analysis across these policies reveals that each either perpetuated or failed to ameliorate racial gaps in opportunities. These policies as implemented, but not as written, disproportionately aided White Americans over Black Americans; or as is the case in policies instituted more recently (e.g., Affordable Care Act), benefited all, but did not address past harms to the Black population.

#### Access to care

The Hospital Survey and Construction Act of 1946 (Pub. L. 79–725), also known as the Hill-Burton Act, required that health care facilities provide care without regard to race. However, the act allowed for separate facilities for White and Black individuals if they provided similar quality services — a provision that was not actively enforced resulting in more money per capita being spent on care for White versus Black residents in Southern states [27]. It was only with Title VI of the 1964 Civil Rights Act (Pub. L. 88–352) that equal access to health care facilities for all races was established, as compliance was tied to hospital funding through the 1965 creation of Medicare. However, private practice physicians and nursing homes were not subject to Title VI integration [27].

#### Education

The GI Bill of 1944 (Pub. L. 78–346) provided all returning World War II veterans generous financial benefits for purchasing a home, enrolling in job training, or attending vocational school or college. While race neutral at face value, to gain the support of Southern Democrats, the administration of the education portions of the bill were left to the states, rather than to a federal agency [28]. So, while the GI Bill did allow many White veterans to improve their job prospects, education, and housing, state- and local-level discriminatory policies, unchecked by the federal government, prevented Black veterans from doing so at the same rate [28].

On May 17, 1954, the U.S. Supreme Court held in the case of *Brown v. Board of Education* (347 U.S. 483 (1954)) that segregating schools by race was a violation of the Equal Protection Clause of the 14th Amendment of the Constitution, overturning previous rulings that allowed for segregated schools so long as the facilities were “separate but equal.” Justice Earl Warren wrote for the majority that separate facilities for Black and White children were inherently unequal (p. 495). Yet, more than 60 years later, school segregation remains prevalent, even if not sanctioned by law, due to persistent neighborhood-level segregation [17, 29].

#### Housing

As part of the New Deal, the National Housing Act of 1934 (Pub. L. 73–479) revamped home mortgages, creating the Federal Housing Administration (FHA), which subsidized home ownership through low-interest mortgages. The FHA relied on the Home Owners Loan Corporation to establish a nationwide property appraisal system to determine eligibility. In addition to local laws or restrictive covenants that prevented Black individuals from purchasing a home in White or “low-risk” neighborhoods, the Home Owners Loan Corporation formalized the process of redlining in which neighborhoods deemed high-risk (predominately Black communities) were not eligible for a mortgage, effectively denying most Black individuals the opportunity to use enabling federal policies to gain home ownership and build wealth at a time when White individuals were supported to do so [30]. The practice of redlining officially ended in 1968 with the passage of the Fair Housing Act (enacted as Title VIII of the Civil Rights Act of 1968; Pub. L. 90–284).

### Population-level outcomes

#### Access to health care

##### Uninsurance rates

**Fig. 1:**
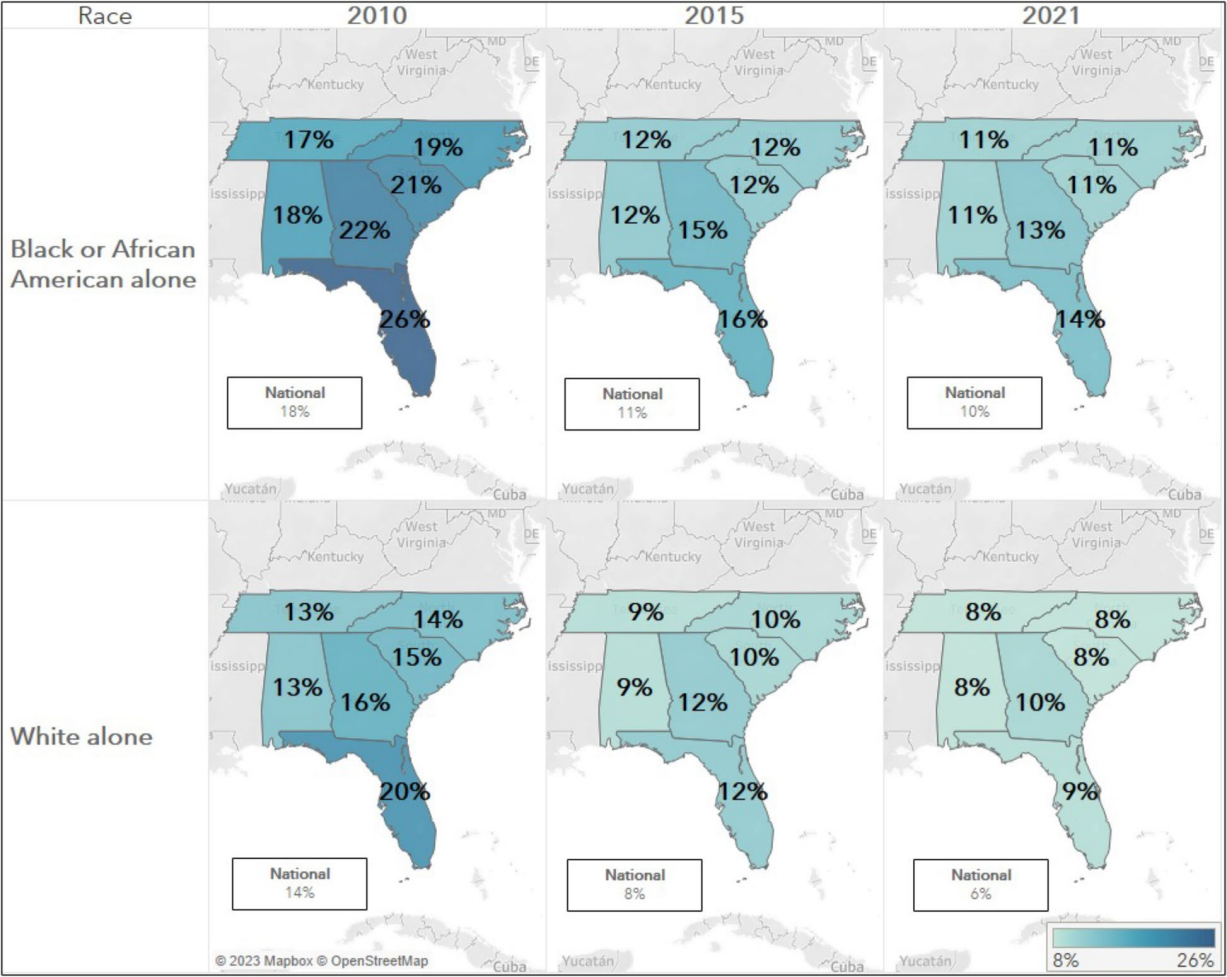
Uninsurance Rate by State versus Nationally by Race, 2010–2021 Note: Black or African American Alone = Black, Not Hispanic and White alone = White, Not Hispanic

Between 2010 and 2021, the uninsured rate dropped in Georgia from 22 to 13% for Black individuals and from 16 to 10% for White individuals. Similar declines were seen across neighboring states and nationally for both races. However, at the state, regional, and national level a racial gap in insurance status still exists, with Black populations having higher uninsurance rates compared to White populations (Fig. 1). Temporally, percent change of decline in uninsured rate were greater in the years immediately following the passage of the Affordable Care Act (Pub. L. 111–148), compared to more recent years (Table 2).

**Table 2:** Percent change in uninsurance rate by state versus nationally by race, 2010–2021

Race	State	2010	2015	2021
**Black or African American alone**	Alabama		-6%	-1%
	Florida		-10%	-2%
	Georgia		-7%	-2%
	North Carolina		-7%	-1%
	South Carolina		-9%	-1%
	Tennessee		-5%	-1%
	United States		-7%	-1%
**White alone**	Alabama		-4%	-1%
	Florida		-8%	-3%
	Georgia		-4%	-2%
	North Carolina		-4%	-2%
	South Carolina		-5%	-2%
	Tennessee		-5%	-1%
	United States		-6%	-2%

Black or African American Alone = Black, Not Hispanic and White alone = White, Not Hispanic

##### Provider shortages

**Fig. 2:**
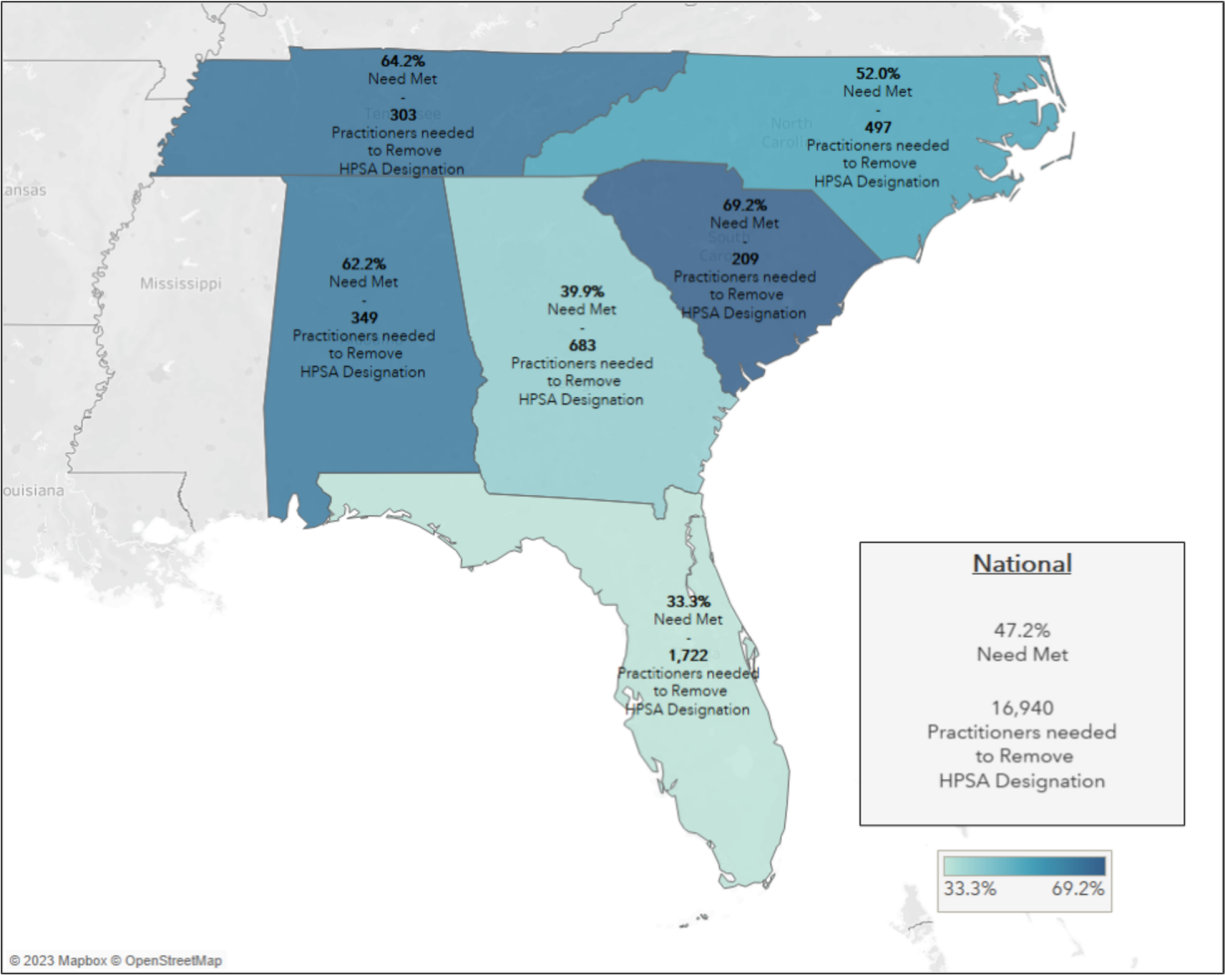
Primary Care Designation Health Provider Shortage Areas by State (based on HRSA data available April 2023 [13])

Using data available in 2023 (HRSA county-level updates 2018 to 2022), Georgia only has 39.9% of provider need met and would need 683 additional practitioners to remove the primary care HPSA designation at the state level. Compared to neighboring states (Fig. 2), only Florida has lower need met (33.3%), with other states having 52.0% to 69.2% of primary care provider need met. The region generally outperforms the country, but Georgia has a lower level of primary care provider need met versus the nation, as a whole (47.2%).

**Fig. 3:**
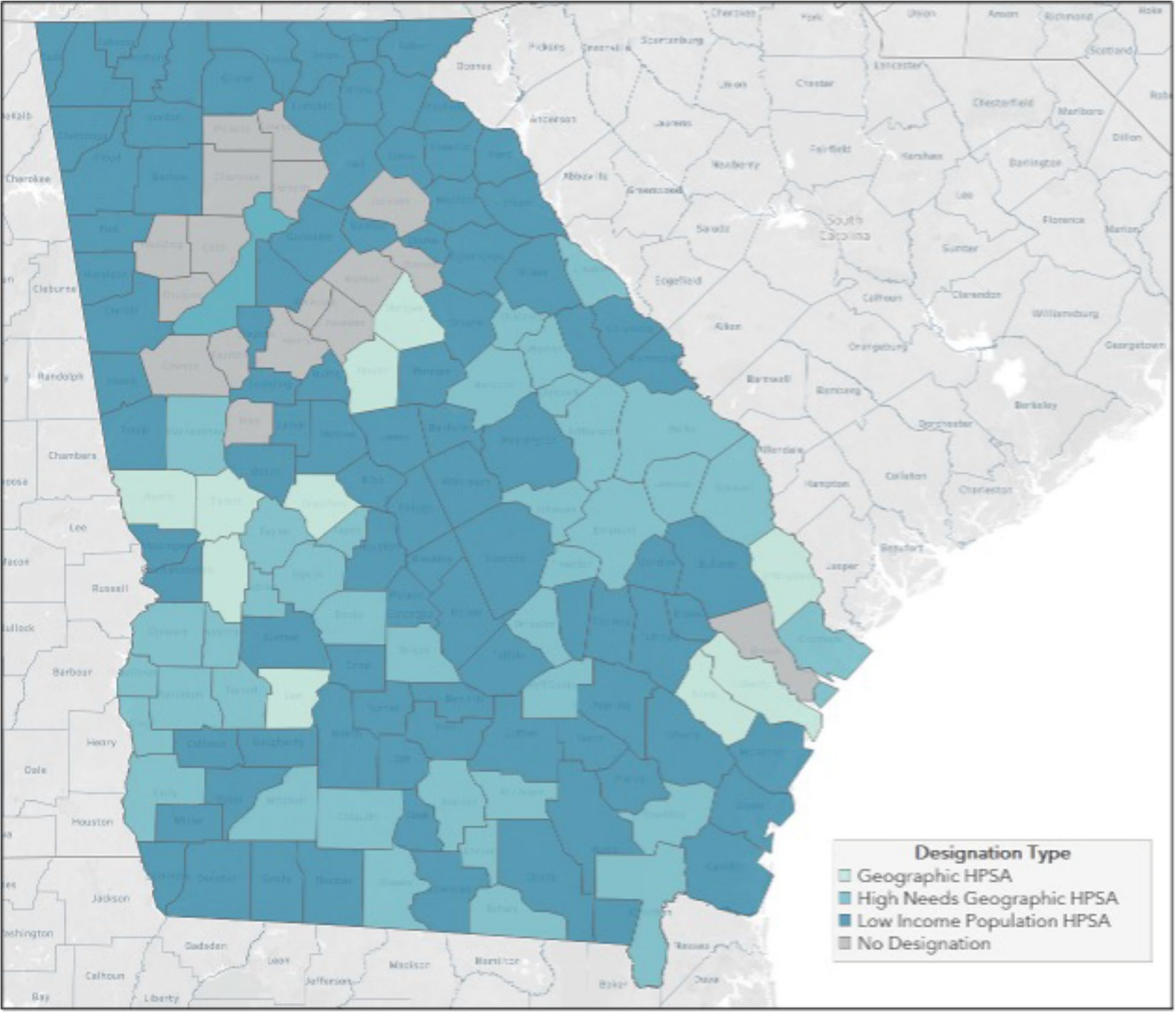
Primary Care Health Provider Shortage Area by Georgia County (based on HRSA data available April 2023 [13]) Note: Designation type based on HRSA HPSA definitions [13]

Specifically, within the state of Georgia, three different types of primary care shortages were identified (Fig. 3). Of the state’s 159 counties, 17 counties have no provider shortages and 10 are classified as geographic need (shortage of providers for an entire group of people within a defined geographic area [13]). Most counties’ shortages stem from provider need to serve the low-income population (*n* = 93 counties), while 39 counties are designated as high needs geographic areas [13].

#### Education

##### Educational attainment

The percent of the overall population with at least a high school degree or higher increased over the past decade for each state and the nation, as well as for both the Black and the White population at each geographic level (Table 3). However, despite this increase in education attainment, a gap still exists between White and Black populations, with Black populations consistently having a lower rate of attainment than the White population in every state and nationwide (Table 3).

**Table 3:** Percent of population with high school degree equivalency or higher by state and race, 2010–2021

State	Race	2010	2015	2021
Alabama	Black or African American Alone			
	White alone			
Florida	Black or African American Alone	78%	82%	85%
	White alone	84%	87%	90%
Georgia	Black or African American Alone	78%	82%	86%
	White alone	87%	89%	94%
North Carolina	Black or African American Alone	82%	85%	89%
	White alone	87%	88%	92%
South Carolina	Black or African American Alone	77%	81%	86%
	White alone	87%	89%	92%
Tennessee	Black or African American Alone	81%	84%	88%
	White alone	85%	87%	91%
United States	Black or African American Alone	82%	85%	88%
	White alone	88%	89%	94%

Black or African American Alone = Black, Not Hispanic and White alone = White, Not Hispanic

##### Public K-12 school demographics

**Fig. 4:**
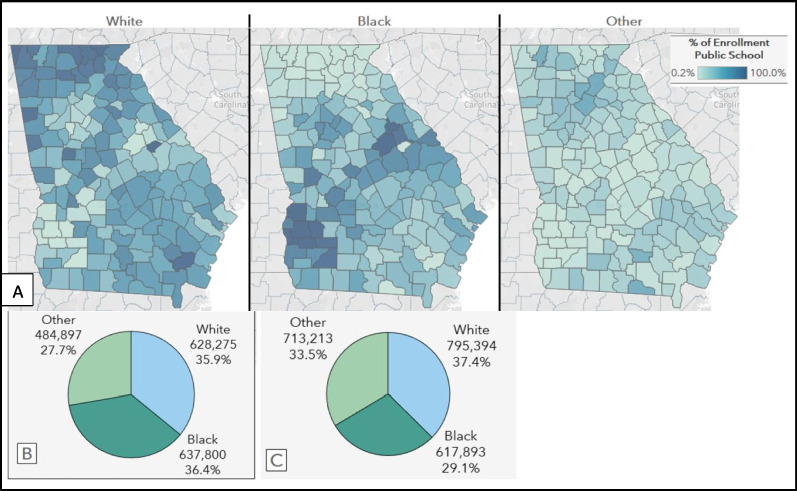
Georgia Public School Racial Composition **A**. Georgia statewide public-school population by race, 2022–2023 academic year **B**. Georgia school-aged children (ages 5 to 17 years) overall by race **C**. Public school enrollment by Georgia county by race, 2022–2023 academic year Note: Other = not White or Black, non-Hispanic

Overall, the race of students enrolled in Georgia public schools (kindergarten through 12th grade) is similar for Black and White students at the start of the 2022–2023 academic school year, although this composition does not reflect racial composition of the state population, overall (Fig. 4). Black children are disproportionately enrolled in the state’s public schools, compared to their makeup of the state population overall.

Most north and southeast Georgia county public schools have predominantly White student populations, while public schools in a band of counties from southwest Georgia to mid-east Georgia have predominantly Black student populations, corresponding to what has been recognized as the “Black Belt” [31] (Fig. 4). Despite general overrepresentation of Black students in Georgia public schools, this distribution is reflective of variance in county-level racial composition.

#### Housing

##### Mortgage and rental burden

Black families have higher rates of mortgage burden (mortgage > 30% of monthly income) versus White families at each time point, in each state, and nationally (Table 4). Regionally, the Black mortgage burden was highest in Florida at each time point (Table 4). Similarly, Black individuals carry higher rental burden (rent > 30% of monthly income) than White individuals in every state and nationally (Table 4).

**Table 4:** Percent of state population with high mortgage or rental burden by state by race, 2010–2021

Race	State	Mortgage Burden			Rental Burden		
		**2010**	**2015**	**2021**	**2010**	**2015**	**2021**
**Black or African American alone**	Alabama	44%	36%	34%	61%	57%	60%
	Florida	59%	45%	41%	68%	63%	66%
	Georgia	49%	37%	30%	61%	57%	57%
	North Carolina	45%	38%	33%	59%	57%	56%
	South Carolina	44%	38%	33%	60%	56%	56%
	Tennessee	45%	37%	30%	59%	55%	55%
	United States	48%	39%	36%	60%	58%	58%
**White alone**	Alabama	30%	22%	21%	50%	45%	43%
	Florida	47%	34%	30%	59%	55%	55%
	Georgia	34%	25%	22%	49%	44%	45%
	North Carolina	32%	25%	22%	48%	45%	45%
	South Carolina	31%	26%	23%	50%	46%	47%
	Tennessee	31%	25%	23%	50%	46%	46%
	United States	36%	28%	25%	51%	48%	48%

Black or African American Alone = Black, Not Hispanic and White alone = White, Not Hispanic

Temporally, each state experienced a decrease in the percent of population (for each the Black and White population) experiencing a high mortgage burden between 2010 and 2021 (Table 5). Georgia’s Black population experienced the largest decrease in mortgage burden (-19%), compared to both neighboring states and the national experience. However, the lessening of the mortgage burden for both the Black and White populations was roughly similar, indicating that there was little impact on reducing the gap between the populations. Over the same period, there was less change in rental burden by population, which was similar in Georgia, regionally, and nationally (Table 5).

**Table 5:** Percent Change in High Mortgage or Rental Burden by State by Race, 2010–2021

Race	State	High Mortgage Burden			High Rental Burden		
		**2010**	**2015**	**2021**	**2010**	**2015**	**2021**
**Black or African American alone**	Alabama		-8%	-2%		-4%	3%
	Florida		-14%	-4%		-5%	3%
	Georgia		-12%	-4%		-4%	0%
	North Carolina		-7%	-5%		-2%	-1%
	South Carolina		-6%	-5%		-4%	0%
	Tennessee		-8%	-7%		-4%	0%
	United States		-9%	-3%		-2%	0%
**White alone**	Alabama		-8%	-1%		-5%	-2%
	Florida		-13%	-4%		-4%	0%
	Georgia		-9%	-3%		-5%	1%
	North Carolina		-7%	-3%		-3%	0%
	South Carolina		-5%	-3%		-4%	1%
	Tennessee		-6%	-2%		-4%	0%
	United States		-8%	-3%		-3%	0%

Black or African American Alone = Black, Not Hispanic and White alone = White, Not Hispanic

##### Homeownership

**Fig. 5:**
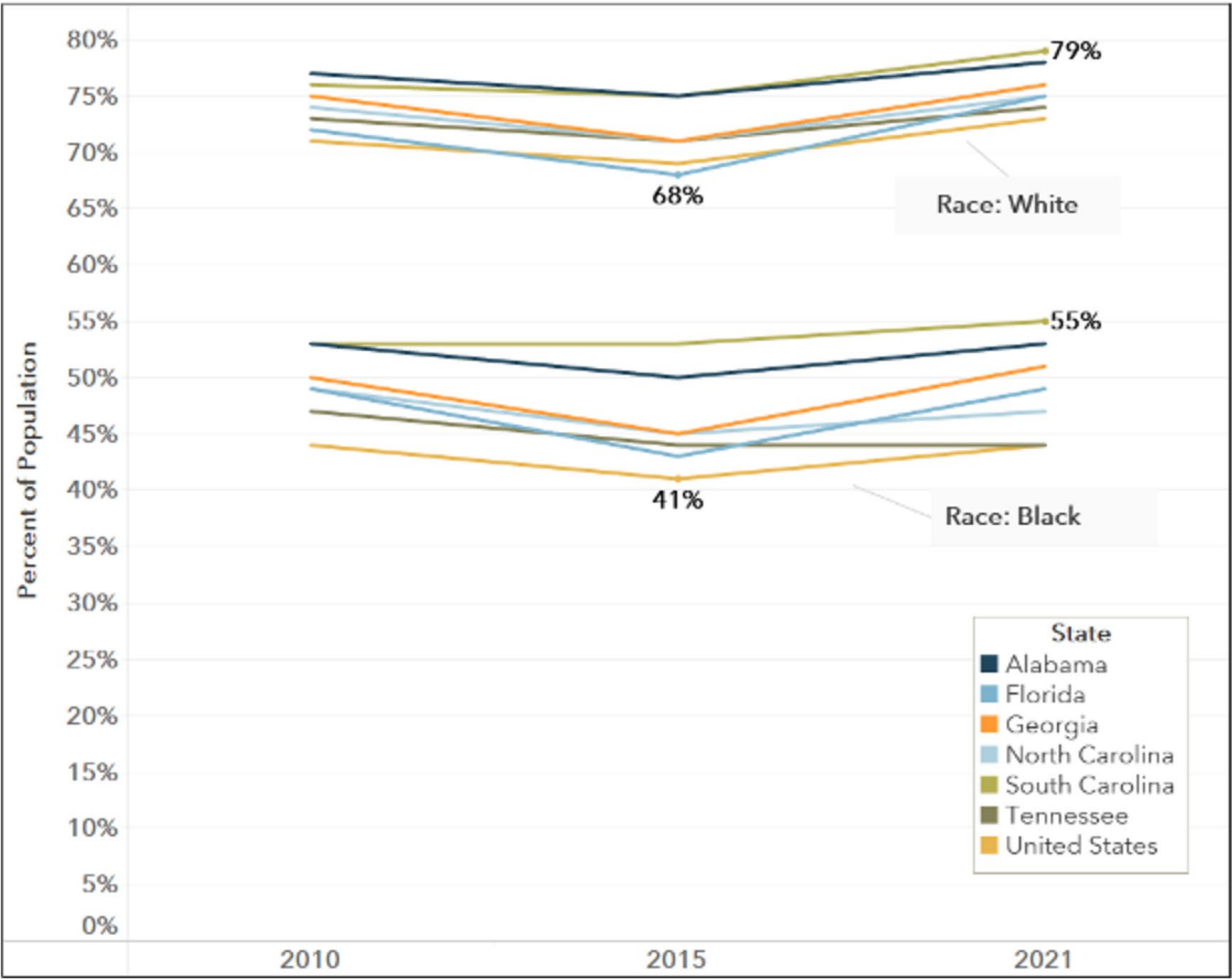
Homeownership Rate by State by Race, 2010–2021

In Georgia, regionally, and nationally, home ownership rates are lower for the Black population versus the White population, across time (Fig. 5). Temporal trends in homeownership rates were comparable across races — dipping in every state across race in 2015, but then increasing, except for the Black population in Tennessee (Fig. 5). However, a large gap persists between Black and White populations in each state and nationally (Fig. 5). Regionally, the Black population has homeownership rates of over 50% in three of the states in 2021, while homeownership rates exceeded 68% for the White population in all six states and nationally.

## Discussion

This analysis adds to a growing body of evidence that structural barriers hamper Black populations’ opportunities to build wealth, gain a quality education, own a home in a neighborhood of opportunity, and access health care, compared to their White peers. This pattern holds true in Georgia, regionally in the Southeast, and nationally in the United States, across multiple measures of social determinants of health.

Some notable population-level outcome patterns emerged from our analyses of social determinants of health. State, regional, and national population-level improvements in health insurance rates, mortgage and rental burden, and educational attainment have occurred over the past decade, with improvements seen for both Black and White populations. However, provider shortages, impacting access to care; rates of home ownership; and school segregation persist.

Seemingly meaningful gaps between the Black and White populations across social determinants of health have not been eliminated at any geographical level. The Black population continues to fare worse than their White counterparts across access to care, housing, and education in all states examined. Comprehensive federal policies, when enforced, appear to have a notable positive, proximal impact (e.g., the Affordable Care Act on insurance rates, *Brown vs. Board of Education* on integration of schools, the Fair Housing Act on homeownership). Yet, these impacts may be insufficient to address long-standing racial gaps given the complexity of shared state and federal governing responsibility in a federalist system and any impact of federal policy may wane over time.

The current reality is that access to U.S. health care is limited by ability to pay. Black individuals are more likely to be uninsured, covered by Medicaid, or be without employer-sponsored health insurance, and less likely to afford health insurance or to pay out-of-pocket for health care [9]. Implementation of the Affordable Care Act, with its expansion of Medicaid and other health insurance options for low- and moderate- income people, is credited with large gains in coverage across all population groups, although a racial coverage gap remains with the uninsured rate for Black Americans at 12% versus 9% for White Americans, overall, [32], and particularly for Black and Hispanic individuals living in non-Medicaid expansion states [33].

Although the practice of redlining officially ended in 1968 with the passage of the Fair Housing Act (enacted as Title VIII of the Civil Rights Act of 1968), a pattern of racially based, geographic residential segregation was entrenched and persists to the present day [30]. A recent report found that 83% of redlined neighborhoods in the 1930s were still highly segregated neighborhoods of color in 2010 [34]. Additionally, more than 50 years after the passage of the Fair Housing Act, home ownership rates for Black individuals remain the lowest of any racial group in the United States (44.1% versus 74.5% for the overall White population in 2020) [35] with this gap seen even for high income earners, despite legislation intended to remedy legal discrimination in the housing market [36].

While federally initiated school desegregation efforts in the 1960s yielded a sharp decline in the percentage of Black students attending predominantly (90%-100%) or mostly (50%-100%) minority schools, there has been an uptick in segregated schools in more recent years [37]. This reversal suggests that a once-and-done approach to policy and federal-level only policy remedies to racial disparities will not have sufficient longevity to create sustained social change.

This uptick in school segregation is driven by neighborhood segregation, which further reinforces an unequal distribution of resources across schools and school districts [18, 38]. In most states, schools are financed through local property taxes, meaning that schools in wealthier districts have more money allocated per student than schools in poorer districts, [18] which is further exacerbated by the tendency for wealthier schools to also receive large amounts of supplemental funding in the form of private donations [39]. Because schools with high rates of poverty often have more minority students, many Black students attend schools that are limited in the services they can provide to students most in need, which can, in turn, drive achievement gaps that persist across all levels of education [38, 40, 41].

Research shows this gap in educational achievement between Black and White students limits not just educational trajectories and career opportunities for many Black children later in life, but also compounds and perpetuates other disparities (e.g., wealth and health) [38]. Studies show that Americans with less education have shorter lives, worse health, more risk factors for disease, and greater rates of disability than those with more education [42].

While we attempt to identify the origins and continued impacts of structural barriers to equity, there are some inherent challenges, as a direct causal relationship is not possible to determine given the interconnectedness of multiple measures of socioeconomic opportunity and generational outcomes. Where one goes to school is determined by where one lives, which is determined by housing policy, income, and preference, which in turn, drives educational opportunity. Thus, the impact of the policy is not linear within in one social sphere, but rather multidimensional and influenced by multiple competing socioeconomic factors, including poverty.

### Using local, nonlegal policy levers to complement federal efforts

Given that the root causes of health disparities and inequities lie at the intersection of health, health care, economics, education, and other social systems, a multisectoral approach is needed to address these systemic issues, including at the structural level [43]. Multisectoral approaches are feasible to inform policy and practice [44], although in a federalist system where governing responsibilities are shared across levels of government, policy or interventions at any one level — local, state, or federal — may provide momentum for proximal benefits for social change, but each alone is insufficient to address needed local system change. Alignment of local, state, and federal policy is needed to address persistent racial disparities in social determinants of health and health outcomes. Research shows that evidence-based, strategic alignment of policy and investment across local, state, and national policy jurisdictions maximizes resources and strengthens outcomes [45]. Further, nonlegal policy interventions, implemented at the local programmatic level, may serve as a tailored, complementary mechanism to address the lingering effects of barriers to equal opportunity.

Thomas, et al. proposed a conceptual framework to achieving health equity that recognizes that detecting disparities, understanding factors driving disparities, and providing evidence-based solutions remains insufficient to eliminate disparities [46]. The results of the fourth generation of health disparities research, they propose, requires utilizing comprehensive, community-based multilevel interventions to address race, racism, and structural inequalities[46].

The following emerging innovations have a supportive evidence base and meet Thomas’ fourth-generation drive for community action that policymakers may consider in developing solutions to break the cycle of racial inequality and meaningfully improve wealth, health, and well-being of historically disadvantaged populations.

#### Overcoming generational poverty

Policies promoting economic equity may have broad health effects. To address income inequality and related health disparities, U.S. cities and states are undertaking many initiatives to improve economic stability and mobility, especially for people of color, including direct cash assistance and wealth-generating programs.

Research shows that direct cash assistance can not only alleviate daily financial challenges and poverty, but can also improve educational, behavioral, health, and development outcomes for both the adults receiving the cash transfer payments and their children [47].

Mayor Michael D. Tubbs (Stockton, Calif.) and the Economic Security Project founded Mayors for a Guaranteed Income in June 2020 [48]. The movement is based on Tubbs’ Stockton Economic Empowerment Demonstration, which gave 125 residents $500 per month for 24 months [49]. An evaluation of this demonstration program showed that the guaranteed income reduced month-to-month income volatility, and recipients reported less depression and anxiety and enhanced well-being. Receipt of guaranteed income also was associated with greater growth in full-time employment [49].

Individual development accounts are a strategy to reduce poverty and build wealth. According to the U.S. Office of the Comptroller of the Currency, individual development accounts are matched savings accounts that enable low-income and low-wealth individuals to save for specified goals within defined timeframes, with limitations on withdrawals and are similar in concept to matched 401(k) plans [50]. The Oregon IDA Initiative was created in 1999 by the state legislature. To date, more than 16,000 Oregonians have participated collectively saving $33 million with $62 million in matching funds distributed [51]. Top saving goals include education (24%), home purchase (22%), microenterprise (18%), and a vehicle (18%) [52]. Evaluation data suggests that Individual development account savers generate continued economic returns with 86% of home buyers making all mortgage payments in the year after purchase, 88% of Individual development accounts -supported businesses still operational after one year, and 71% of education savers completing or still enrolled in postsecondary education [52].

Community land trusts are a strategy to provide long-term affordable home ownership opportunities to low- and middle-income families with the goal of helping these families build wealth while growing affordable housing options [53]. One-time public or private investment is used to acquire or build a home. The home is sold to a low- or middle-income buyer, while the trust retains ownership of the land. The community land trust manages future sales of the home ensuring that the home is sold at an affordable rate to a future family, while the current homeowner realizes some wealth gain from the sale.

### Limitations

Limitations of this study should be noted, particularly in two areas. First, there were limitations in data selection, availability, and quality. The authors did their best to identify publicly available data that was both associated with a comprehensive piece of federal legislation meant to alleviate inequities resulting from long-standing structural barriers and had an evidence base supporting its use as a proxy for a social determinant of health. However, there was no one publicly available data source that covered all identified social determinants of health. Thus, comparisons of trends across social determinants of health may be limited. Further, not all data was available for each desired time period or across geographies (e.g., school segregation), so some comparisons were limited. Specific to school segregation in Georgia, the authors acknowledge that there can be variation in segregation of individual schools based upon neighborhood-level composition that may not be captured with county-level data. While the authors would ideally have been able to assess historical trends dating back to the time each piece of federal policy was implemented, such data was not available, which necessitated the use of contemporary data, thus hampering the ability to precisely measure more proximal impact of federal policy in the Southeast. Lastly with regard to data, the authors acknowledge that other disadvantaged populations, besides Black populations, exist and have been harmed by structural barriers to opportunity. However, racial breakdowns in data beyond Black and White populations were incomplete or noncomparable, and thus, were not included in this analysis.

Another area of limitations is the interconnectedness of social determinants of health. While the authors attempted to find proxy measures for the three key social determinants of focus to assess trends, the authors acknowledge that in the real world, social determinants are highly interrelated and do not exist in isolation. Similarly, policy — even landmark federal policy — does not operate in isolation and no direct causal relationship or even direct correlation can be inferred decades later at the population level from each individual policy examined. Thus, the identified trends should be interpreted with caution given the multifactorial nature of health, poverty, housing, and educational outcomes. Impact on ultimate population-level outcomes, like life expectancy, would be even more challenging to extrapolate given the interconnectedness of social determinants of health.

Given these limitations, and the inherent impact shortcomings of federal-only policy in a federalist system, the authors suggest that future research should examine how local, state, and federal policies interact to support sustainable change in outcomes. The field would also benefit from cohort studies tracking change in socioeconomic status and health outcomes over time, particularly for locally implemented policy or complementary programmatic interventions.

## Conclusion

Seemingly meaningful gaps between the Black and White populations across social determinants of health (access to care, education, and housing) have not been eliminated in Georgia, the Southeast region, or nationally in the United States. This analysis adds to a growing body of evidence that historically racialized social structures hamper Black populations’ opportunities to build wealth, gain a quality education, own a home in a neighborhood of opportunity, and access health care, compared to their White peers. While federal laws do provide momentum for proximal benefits for social change, they alone are insufficient to address needed local system change and nonlegal policy interventions, implemented at the local programmatic level, may serve as complementary mechanism to address the lingering effects of barriers to equal opportunity, and ultimately, health equity.

## Data Availability

The datasets used and/or analyzed during the current study are available from the corresponding author on reasonable request.

## Abbreviation

HPSA: Health Professional Shortage Areas
HRSA: Health Resources and Services Administration
FHA: Federal Housing Administration
